# Spatio-temporal distribution characteristics and influencing factors of COVID-19 in China

**DOI:** 10.1038/s41598-021-83166-4

**Published:** 2021-02-12

**Authors:** Youliang Chen, Qun Li, Hamed Karimian, Xunjun Chen, Xiaoming Li

**Affiliations:** 1 School of Civil and Surveying and Mapping Engineering, Jiangxi University of Science and Technology, Ganzhou, China; 2 School of Information Engineering, Jiangxi University of Science and Technology, Ganzhou, China; 3 Department of Spinal Surgery, Ganzhou People’s Hospital Jiangxi, Ganzhou, China; 4 School of Geosciences and Info Physics, Central South University, Changsha, China

**Keywords:** Sustainability, Epidemiology

## Abstract

In December 2019, corona virus disease 2019 (COVID-19) has broken out in China. Understanding the distribution of disease at the national level contributes to the formulation of public health policies. There are several studies that investigating the influencing factors on distribution of COVID-19 in China. However, more influencing factors need to be considered to improve our understanding about the current epidemic. Moreover, in the absence of effective medicine or vaccine, the Chinese government introduced a series of non-pharmaceutical interventions (NPIs). However, assessing and predicting the effectiveness of these interventions requires further study. In this paper, we used statistical techniques, correlation analysis and GIS mapping expression method to analyze the spatial and temporal distribution characteristics and the influencing factors of the COVID-19 in mainland China. The results showed that the spread of outbreaks in China’s non-Hubei provinces can be divided into five stages. Stage I is the initial phase of the COVID-19 outbreak; in stage II the new peak of the epidemic was observed; in stage III the outbreak was contained and new cases decreased; there was a rebound in stage IV, and stage V led to level off. Moreover, the cumulative confirmed cases were mainly concentrated in the southeastern part of China, and the epidemic in the cities with large population flows from Wuhan was more serious. In addition, statistically significant correlations were found between the prevalence of the epidemic and the temperature, rainfall and relative humidity. To evaluate the NPIs, we simulated the prevalence of the COVID-19 based on an improved SIR model and under different prevention intensity. It was found that our simulation results were compatible with the observed values and the parameter of the time function in the improved SIR model for China is a = − 0.0058. The findings and methods of this study can be effective for predicting and managing the epidemics and can be used as an aid for decision makers to control the current and future epidemics.

## Introduction

In December 2019, a case of typical pneumonia [novel coronavirus disease 2019 (COVID-19)] was found in Wuhan, Hubei province in China^1,2^. COVID-19 is transmitted in various ways, mainly respiratory droplets and close contact transmission^3–5^. Infected patients and asymptomatic infections^6,7^ are the main source of infection. The disease is highly contagious, and can spread without symptoms in a short period of time, resulting in a pandemic.

On March 12, 2020, the virus has spread to all provinces of China and some other countries. China has a total of 80,813 confirmed cases and 3176 deaths. In the absence of specific medicine and vaccine to counter the virus, non-pharmaceutical interventions (NPIs) play an important role to control epidemics. Bo et al.^8^ divides the implemented NPIs into four categories: mandatory face mask in public, isolation or quarantine, social distancing and traffic restriction. There have been several researches to evaluate the effectiveness of these NPIs. However, most of them focused on assessing the impact of individual NPIs on cities and countries^9–11^. To control an epidemic, many NPIs are carried out simultaneously. Therefore, assessing individual NPIs, without considering other interventions, may not give a right view related to effectiveness of NPIs. In order to control the outbreak, the Chinese government has introduced a series of prevention and control measures, such as extending the Spring festival holiday, postponing the resumption of work and school, traffic control and calling for home isolation measures, in both national and local level. Moreover, districts across the country were closed, restrictions on travel and crowd gathering were implemented and the strict body temperature check was carried out in public places. However, the contribution of these policies to control the spread of COVID-19 needs to be further evaluated.

Some studies have investigated the relationship between COVID-19 and meteorological factors. However, there are discrepancies between their conclusions. Pan et al.^12^ studied 202 sites in 8 countries and claimed that the meteorological factors had no significant effect on the spread of COVID-19. The findings from studies in Spain^13^ and Iran^14^ were also have had the same results. However, other studies came to an opposite conclusion that meteorological factors, such as temperature and relative humidity, were associated with COVID-19 cases. Tosepu et al.^15^ used Spearman method to analyze the correlation between weather and COVID-19 pandemics in Jakarta, Indonesia. They found that only the average temperature was significantly associated with the COVID-19 pandemic. Menebo et al.^16^ analyzed the correlation between metrological factors and COVID-19 prevalence in the Norwegian capital Oslo and found that the highest temperature and normally mild precipitation have influence on distribution of the virus. Some studies also get the result that temperature was related to the COVID-19 epidemic^17–20^. From above, it can be inferred that the results on the effects of meteorological conditions on the spread of COVID-19 remain controversial. Consequently, further analysis is necessary.

Since the outbreak of the COVID-19 epidemic, various scholars have carried out research on it, especially in the medical field, mainly focused on epidemiology^21,22^, pathology^23^ and pharmacology^24–26^. However, the discovery of vaccine for this virus is a relatively long process. It is estimated that most epidemiological studies have space–time properties, and the effective use of space–time attributes can help people to understand the disease more fully. Spatial and temporal spread of COVID-19 has been recently reported for Italy^27^, US^28^, Spain^13^ and Bangladesh^29^. In China, Liu et al.^30^ investigated the spatial and temporal characteristics of nighttime light radiance and air quality index before and during the pandemic in mainland China and concluded that the outbreak and spread of COVID-19 had a crucial impact on people’s daily lives, and activity ranges through the increased implementation of lockdown and quarantine policies. Kang et al.^31^ described the spatiotemporal pattern and measured the spatial association of the early stages of the COVID-19 epidemic in mainland China from 16 January to 6 February 2020. Their results showed that the COVID-19 infections had a significant spatial association. Xiong et al.^32^ used spatial autocorrelation and Spearman’s rank correlation methods to investigate the spatiotemporal patterns and influencing factors of the COVID-19 epidemic in Hubei province. Until now, studies evaluating the spatial spread of the COVID-19 pandemic and its influencing factors in national level in China are limited. Xie et al.^33^ analyzed the temporal and spatial differentiation characteristics of the COVID-19 epidemic in mainland China, and used the geographic detector method to detect the correlation between detection factors and the epidemic spread rate. However, for meteorological factors, they only considered temperature and didn’t consider other meteorological factors. Moreover, they did not analyze China’s national control measures.

This study investigates the temporal and spatial distribution characteristics of the COVID-19 outbreak in China. For this purpose, the influence of different meteorological factors and the proportion of the population flow entered from Wuhan on distribution of the virus are explored. Moreover, as one of the important factors in epidemics control is the actions which are done by governments, we analyze the effects of non-pharmaceutical interventions introduced by China after the outbreak of the epidemic. We also predict the number of infected cases under different controlling scenarios and conditions.

## Data and methods

### Data collection

This study used COVID-19 related data of different cities in China from 20 January to 12 March 2020. These data, including daily confirmed cases, cumulative confirmed cases, daily recovered cases and daily deaths numbers of Coronavirus disease were obtained from the website that provides real-time data on COVID-19 outbreak (https://ncov.dxy.cn/ncovh5/view/pneumonia).

The total population data of each province in mainland China were obtained from the “China Statistical Yearbook 2019”. The permanent population data of Macau and Hong Kong came from Sogou Encyclopedia. Since Taiwan collects permanent population data every 10 years, the permanent population of Taiwan in 2019 was predicted by the World Population Encyclopedia website. Population migration data were collected from Baidu Migration-Baidu Maps Smart Eye Platform (https://qianxi.baidu.com/).

Meteorological data during the same study period for each city were collected from Tutiempo.Net (https://en.tutiempo.net/). Meteorological factors included daily average temperature (°C), daily maximum temperature (°C), daily minimum temperature (°C), diurnal temperature variation (°C), atmospheric pressure at sea level (hPa), relative humidity (%), precipitation (mm), average visibility (km) and average wind speed (km/h).

### Methods

#### Spatial autocorrelation analysis

Spatial autocorrelation analysis^34^ includes global spatial autocorrelation and local spatial autocorrelation. The global spatial autocorrelation is generally measured by Moran’s *I* index. The Moran’s *I* index reflects the similarity of the attribute value of interest (here the COVID-19 confirmed cases) in spatially adjacent units. A positive Moran’s *I* (close to + 1) indicates clustering (i.e., low–low or high–high). While negative global Moran’s values (near to − 1) conforms to a dispersed pattern (i.e., high–low–high–low or a checkboard pattern).

Global spatial autocorrelation measures the overall clustering of data and provides only one set of values for entire study area which does not suggest the location of the clusters. On the other hand, local spatial autocorrelation explores within the global pattern to recognize clusters (hot spots) that either drives the overall clustering, or that shows heterogeneities that depart from global pattern. We used the local hot spot analysis tool (Getis-Ord Gi*)^35,36^ to explore the spatial gathering hotspots and cold spots of the COVID-19 epidemic in Chinese cities. The Getis-Ord Gi* Index is calculated through Eq. (1).

1 $$G_{i}^{*} { = }\frac{{\sum\nolimits_{{{\text{j}} = 1}}^{n} {w_{i,j} x_{j} - \overline{x}\sum\nolimits_{j = 1}^{n} {w_{i,j} } } }}{{S\sqrt {\frac{{\left[ {n\sum\nolimits_{j = 1}^{n} {w_{i,j}^{2} - \left( {\sum\nolimits_{j = 1}^{n} {w_{i,j} } } \right)^{2} } } \right]}}{n - 1}} }}$$

where n is the number of spatial units that need to be analyzed, x_i_ and x_j_ are the attribute values of the COVID-19 confirmed case in locations i and j, respectively, W_ij_ is the spatial weight between elements i and j, and $\overline{x}$ is the average value of the attributes and S is the standard deviation.

#### SIR model

The controlling actions are one of the important elements on every epidemic. Therefore, utilizing appropriate method to investigate the efficiency of control measures is necessary. The susceptible–infected–recovered (SIR) model is a classic model for studying the dynamics of infectious diseases. The model was established by Kermack and McKendrick in 1927 using the dynamic method^37^. Based on the SIR model, we analyzed the effects of relevant prevention and control measures done by China during the outbreak by adding new parameters to characterize the changes in infection coefficients over time. The dynamics functions constructed according to the basic characteristics of the SIR model are as following:

2 $$\left\{ {\begin{array}{*{20}l} {\frac{dS}{{dT}} = \frac{ - \beta IS}{N}} \hfill \\ {\frac{dI}{{dT}} = \frac{\beta IS}{N} - \gamma I} \hfill \\ {\frac{dR}{{dT}} = \gamma I} \hfill \\ \end{array} } \right.$$

where S is susceptible population, I is infected population and R is recovered population. N is the total number of people (N = S + I + R). β is the infection factor, which is the average number of people exposed per day by the probability of the contact. Until the COVID-19 epidemic is effectively controlled, the government's prevention and control measures will be continuously strengthened. Consequently, infection factor, which can be characterized by the time function (β = at + b)^38^, continues to decline accordingly. Generally speaking, *a* is a negative value, and the greater the prevention and control efforts are, the greater its absolute value will be. γ is the recovered factor, and it is proportional to the inverse of the average infection time (14 days). As the proportion of the infected and recovered cases to the total population of China is very small in the early period (less than 0.01%), it is ignored here, and we assumed that the susceptible number is equal to the total population. Finally, the Eq. (2) is simplified to the following:

3 $$I = I_{0} {\text{e}}^{(\beta - \gamma )t} \Rightarrow {\text{Ln}}\frac{I}{{I_{0} }} = at^{2} + (b - \gamma )t$$

where I_0_ is the initial number of infected people. Here we define January 20 as the first day with I_0_ = 260. Based on the collected data of China's COVID-19 epidemic, the values of parameters *a* and *b* in Eq. (3) were obtained through non-linear regression analysis performed in Python. After that, the predicted prevalence curve of COVID-19 was obtained in MATLAB. By modifying the value of *a* in the simulation, the prevalence curve of COVID-19 under different infection coefficients was predicted, and the epidemic prevalence under different prevention and control forces was evaluated.

#### Statistical analysis

Correlation analysis refers to the analysis of two or more variables in order to investigate the strength of the relationship between them. Because the data were not normally distributed, we used the Spearman rank correlation to find the relationship between meteorological factors and cumulative COVID-19 cases. The Spearman rank correlation is calculated using the Eq. (4).

4 $$\rho = 1 - \frac{{6\sum {d_{i}^{2} } }}{{n(n^{2} - 1)}}$$

In the above, *d*_*i*_ represents the difference between the ranks of two parameters, and *n* is the number of samples in dataset. *ρ* represents the correlation coefficient between two parameters, the range is [− 1,1].

## Results and discussion

### Spatial and temporal distribution characteristics of COVID-19

#### Temporal distribution characteristics

**Figure 1:**
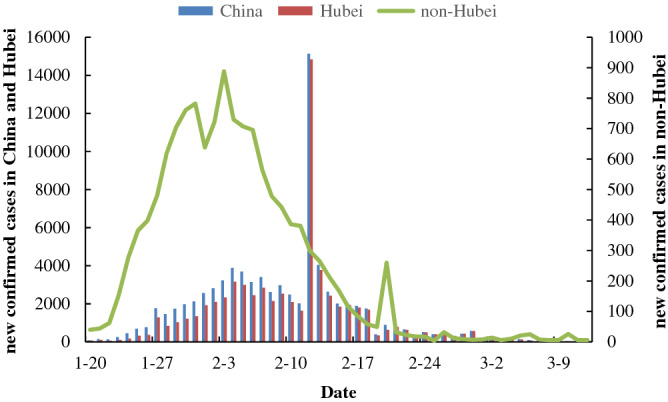
The trend chart of new confirmed cases in China, Hubei and non-Hubei provinces.

Figure 1 illustrates the trend chart of new confirmed cases in China, Hubei and non-Hubei provinces. The results show that the overall trend of daily new confirmed cases in China and Hubei Province was consistent with the time distribution. Since the epidemic outbreak, the first peak of confirmed cases per day was reached on February 4th, followed by a slow decline. On February 12th in line with the report of Hubei province confirmed cases^39^, the highest number of new confirmed cases was observed. This is followed by a continuous decline in the number of new cases. As of March 12, the cumulative cases in Hubei Province accounted for 83.6% of the total number of cases in China. In order to better understanding of the changes in non-Hubei provinces, we divided the daily COVID-19 change curve for non-Hubei provinces into 5 stages: stage I: Before January 22, this phase is the initial stage of the COVID-19 outbreak in which the number of confirmed cases is relatively small, the daily new cases are more moderate; stage II: From January 23 to February 3, due to the spread way of the COVID-19, the disease spread rapidly and the daily new cases reached the first peak on January 31. Then after a slight decline, it increased again and reached the highest rate of the entire study period on February 3; stage III: From 4 to 19 February, the outbreak in this phase was well controlled and the number of new cases continued to decline on a daily basis; stage IV: On February 20, the new confirmed cases in this phase rebounded, mainly due to the large number of confirmed cases in prisons in Zhejiang and Shandong provinces^40^; stage V: From 21 February to 12 March, the outbreak was largely under control, the number of new cases in the single digits by 12 March.

#### Spatial distribution characteristics

**Figure 2:**
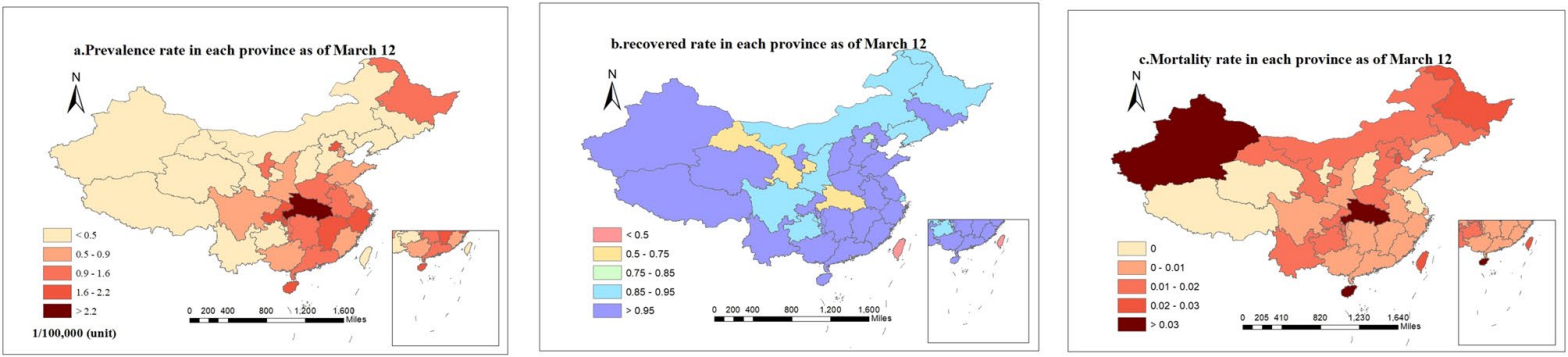
Distribution of the prevalence (**a**), recovered rate (**b**) and mortality rate (**c**) of COVID-19 in China.

Figure 2 illustrates the distribution of prevalence rate, recovered rate and mortality rate of COVID-19 in China. As can be seen, the prevalence rate in 15 provinces, including Hubei, Zhejiang, Beijing, Jiangxi and Chongqing, is higher than 0.9/100,000. The prevalence rate in Hubei is the highest (greater than 2.2/100,000). Moreover, higher prevalence rate was observed among the provinces around Hubei than other provinces (Fig. 2a). Since the case of COVID-19 was first reported in Wuhan in Hubei, and COVID-19 has human-to-human contact infection, it can be inferred that the prevalence of the epidemic is closely related to the distance from Wuhan and it has spatial characteristic. The high prevalence rate in Heilongjiang, that is far from Wuhan, can be explained by the large population flow (due to the tourist season) and the very cold weather that forces the people to keep the windows closed which disturbs the ventilation, and helps virus transmission. At the same time, provinces with high population movements, such as Beijing, Guangdong and Shanghai, have relatively high prevalence rates, while the prevalence rate was lower in the sparsely populated regions in the northwest of China. The recovered rate in all regions of China was relatively high, with the recovered rates above 85% in 29 provinces, and only Beijing, Hubei, Gansu, Hong Kong and Taiwan’s provinces and cities have the recovered rate of less than 85% (Fig. 2b). Mortality rate was relatively low in all regions of China, with zero deaths in the six provinces (Tibet, Qinghai, Shanxi, Jiangsu, Ningxia and Macau). However, the mortality rate of more than 3% was observed in Hubei, Xinjiang and Hainan (Fig. 2c). Due to the high number of confirmed cases in Hubei Province, the limited number of medical facilities and skilled staff, in short supply, resulting in a significantly higher mortality rate. While Xinjiang and Hainan have few confirmed cases, higher mortality rates were observed than in the other regions which may be explained by the relatively backward medical conditions and accidental results.

#### Local spatial autocorrelation

**Figure 3:**
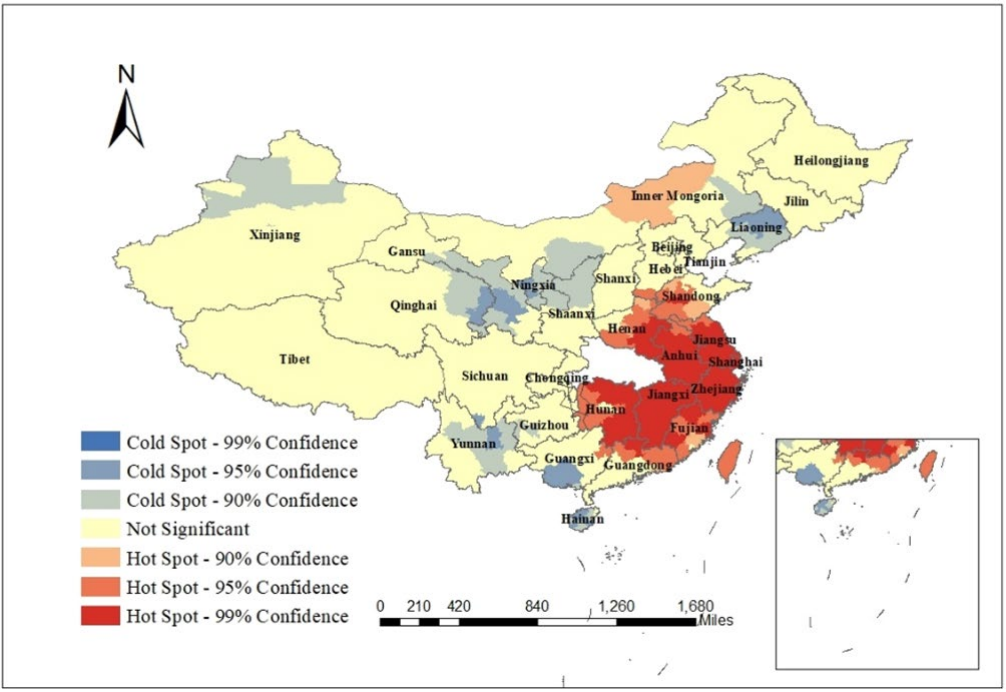
The results of local spatial autocorrelation of cumulative confirmed cases in cities other than Hubei Province in March 12. The map was generated by ArcGIS (http://www.esri.com/software/arcgis/arcgis-for-desktop/free-trial).

Figure 3 shows the results of a hot spot analysis using the Getty-Ord Gi method for cumulative confirmed cases on 12 March. It is worth mentioning that as the new confirmed cases of COVID-19 in Hubei Province were much higher than in other regions, in order to better understand the characteristics, we excluded the data related to Hubei urban units.

As can be seen, the hot spots were concentrated in the cities in the southeast of Hubei Province, Taiwan and the cities in Inner Mongolia (*P* < 0.05). This indicates that there were more confirmed cases in these cities and their surrounding cities. These cities are geographically close to Wuhan with large population flow, and are densely populated. The cold point regions are scattered, mainly in some cities of Qinghai, Gansu, Ningxia, Shaanxi, Inner Mongolia, Liaoning, Xinjiang, Guizhou, Yunnan, Sichuan, Guangxi and Hainan provinces.

#### Spatial–temporal dynamic characteristics

**Figure 4:**
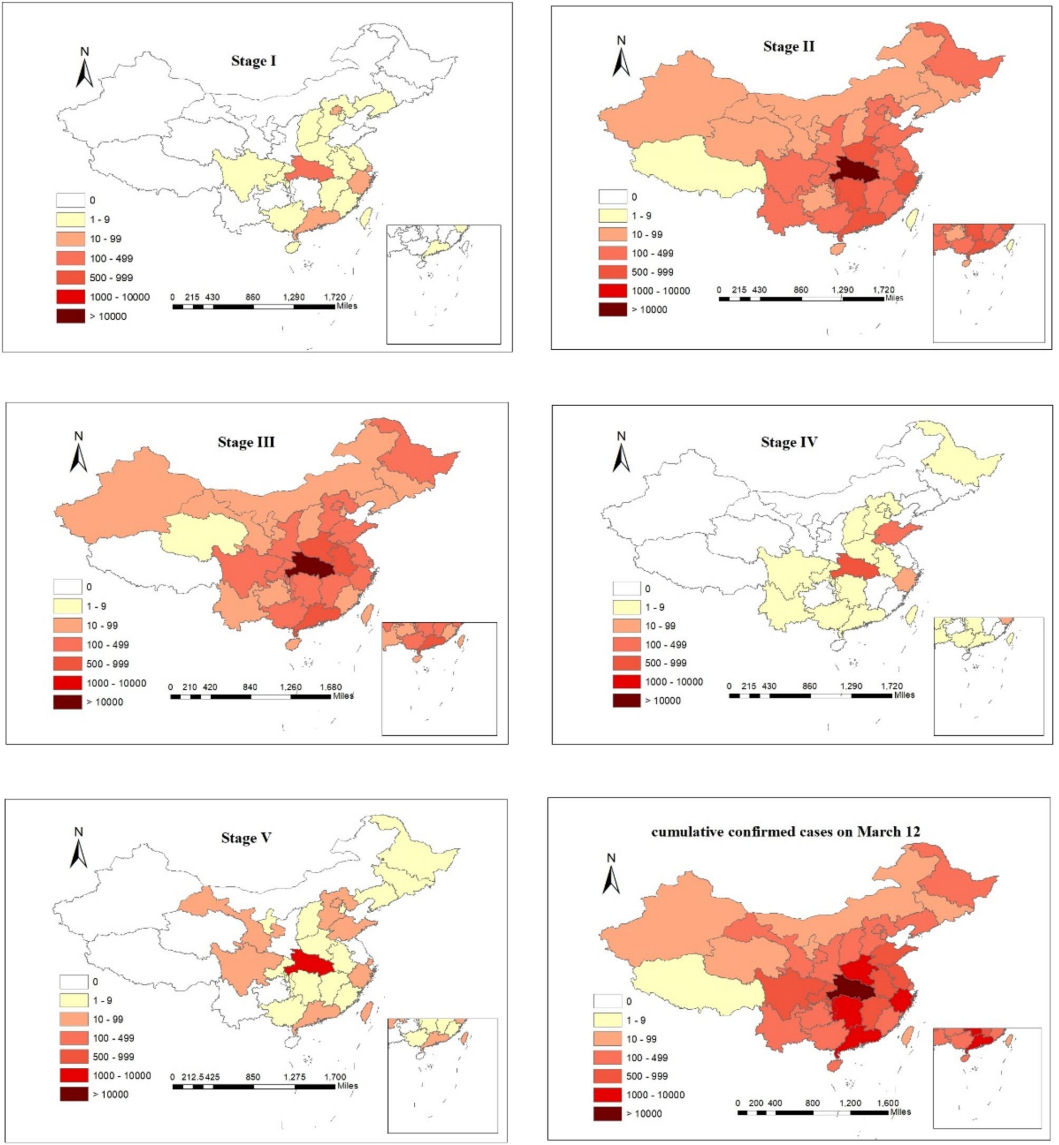
Distribution of newly confirmed cases in China at various stages and cumulative confirmed cases on March 12.

Figure 4 shows the spatial distribution of confirmed cases of COVID-19 in China in each of the mentioned five stages ("Temporal distribution characteristics" section). As can be seen, in all stages Hubei province had the highest number of confirmed cases. During the stage I, confirmed cases were reported, only from 20 provinces, including Hubei, Guangdong, Zhejiang, Jiangxi and Henan, with more than 100 new confirmed cases in Hubei. In the stage II, there were confirmed cases in all provinces of China. More than 100 new cases have been reported in 18 provinces, including Jiangxi, Anhui, Chongqing and Jiangsu. Among them, Hubei, Zhejiang, Guangdong and Henan provinces announced over 500 new cases. In the stage III, Tibet saw zero growth for the first time. There were 17 provinces including Jiangxi, Hunan, Zhejiang and Jiangsu that reported more than 100 newly confirmed cases. Among them, Hubei, Henan, Guangdong and Anhui confirmed over 500 new cases. During the stage IV, most provinces experienced zero growth, and only Hubei and Shandong reported more than 100 new cases. In the stage V, only Hubei Province had above 100 newly confirmed cases. In summary from beginning of the epidemic till March 12, 26 provinces reported above 100 confirmed cases, from which more than 10,000 were reported in Hubei and more than 1000 in Guangdong, Henan, Zhejiang and Hunan. Moreover, the areas with more new confirmed cases are mainly in the southeast. The high population density and population flow in the southeast are the main reasons that accelerated the spread of COVID-19. Consequently, the newly confirmed cases in the southeast are relatively higher than other regions. In contrast with the previous studies (Briz-Redón and Serrano-Aroca 2020^13^; Masrur et al. 2020^29^; Xie et al. 2020^33^) that broke down the epidemic period into smaller segment based on time (e.g. week or month), dividing the epidemic period based on the number of confirmed cases can give us more insightful view about the spatial and temporal dynamic characteristics of the epidemic. Moreover, in the case of dividing the epidemic period based on time, it is difficult to find the specific time of decline or rise in the number of cases.

### Analysis of the influencing factors on distribution of COVID-19

#### Correlation analysis between meteorological factors and COVID-19

Table 1 provides the descriptive statistics for cumulative confirmed cases of COVID-19 and meteorological factors in 31 major cities (27 provincial capitals and 4 municipalities) of China. During the study period, there were a total of 52,994 cases in 31 cities. Due to the considerable difference between the north and south latitude of China (~ 15°), the weather conditions vary significantly across the country. The difference between the highest reported values of average temperature, maximum temperature, minimum temperature, diurnal temperature variation, atmospheric pressure, relative humidity, rainfall, visibility and wind speed and the lowest values during this period were 32.95 °C, 29.87 °C, 37.52 °C, 11.22 °C, 21.25 hPa, 64.76%, 11.9 mm, 22.25 km and 11.06 km/h, respectively.

**Table 1:** Cumulative description of confirmed cases and meteorological factors in 31 major cities.

Variables	Effective	Min	Max	Mean	SD
Total COVID-19 cases	31	1.00	49,122.00	1709.48	8800.44
Average daily COVID-19 cases	31	0.02	1198.10	41.70	214.65
T (°C)	31	− 12.71	20.24	4.50	7.85
TM (°C)	31	− 5.79	24.08	10.71	6.88
Tm (°C)	31	− 20.20	17.32	− 1.42	9.16
TD (°C)	31	6.76	17.98	12.13	3.23
SLP (hPa)	26	1009.83	1031.08	1024.29	4.51
H (%)	31	16.66	81.42	63.84	15.03
PP (mm)	31	0	11.90	1.92	2.60
VV (km)	31	5.89	28.14	10.86	4.50
V (km/h)	31	4.52	15.58	8.74	2.11

*COVID-19* Corona Virus Disease 2019, *Min* minimum, *Max* maximum, *SD* standard deviance, *T* average temperature, *TM* maximum temperature, *Tm* minimum temperature, *TD* diurnal temperature variation, *SLP* atmospheric pressure at sea level, *H* relative humidity, *PP* total precipitation, *VV* average visibility, *V* average wind speed.

Table 2 shows the results of Spearman correlation between the cumulative confirmed cases of COVID-19 and various meteorological factors in 31 cities. As is shown, the correlations between the cumulative confirmed cases in 31 cities and the average temperature, maximum temperature, minimum temperature, diurnal temperature variation, relative humidity and precipitation were statically significant (*P* < 0.01). Relatively high positive correlations were observed between cumulative confirmed cases of COVID-19 and temperature (including daily average temperature, daily maximum temperature and daily minimum temperature), relative humidity and rainfall. This indicates that an increase in either of the mentioned meteorological factors enhances the number of confirmed cases. These results agree with those in Liu et al.^41^ and Mofijur et al.^42^ where the mean temperature has positive correlation with the COVID-19 cases. The positive correlation between number of confirmed cases and relative humidity and precipitation can be explained by this fact that in relatively moist air, the virus can easily stick to the surface of the object and reproduce. This helps the spread of virus into further bodies, human contact with the object was easy to infect the virus. Tamerius et al.^43^ and Park et al.^44^ reported that humid-rainy conditions were associated with seasonal influenza epidemics. The cumulative number of confirmed cases was negatively correlated with the diurnal temperature variation, indicating that the novel coronavirus might be more suited to survive in an environment with small diurnal temperature variation or constant temperature. Some studies are consistent with this result. Liu et al.^41^ has found that each 1 °C increase in the diurnal temperature variation was associated with the decline in number of patients. Lambrechts et al.^45^ has found that larger diurnal temperature variation may prevent dengue virus infection and reduce the risk of transmission.

**Table 2:** Spearman's correlation between COVID-19 and meteorological factors.

	S	T	TM	Tm	TD	SLP	H	PP	VV	V
S	1									
T	0.484**	1								
TM	0.463**	0.987**	1							
Tm	0.493**	0.987**	0.960**	1						
TD	− 0.492**	− 0.752**	− 0.673**	− 0.824**	1					
SLP	− 0.207	− 0.770**	− 0.785**	− 0.739**	0.399	1				
RH	0.530**	0.636**	0.610**	0.683**	− 0.789**	− 0.478*	1			
PP	0.487**	0.453*	0.429*	0.518**	− 0.583**	− 0.210	0.637**	1		
VV	− 0.305	− 0.009	0.006	− 0.002	0.040	− 0.225	− 0.121	0.084	1	
V	0.036	0.185	0.214	0.163	0.003	− 0.333	0.347	0.217	0.101	1

*S* sum of COVID-19 cases, *T* average temperature, *TM* maximum temperature, *Tm* minimum temperature, *TD* diurnal temperature variation, *SLP* atmospheric pressure at sea level, *RH* relative humidity, *PP* precipitation, *VV* average visibility, *V* average wind speed.

**P < 0.01; *P < 0.05.

#### Effect of the population flow on distribution of COVID-19

**Figure 5:**
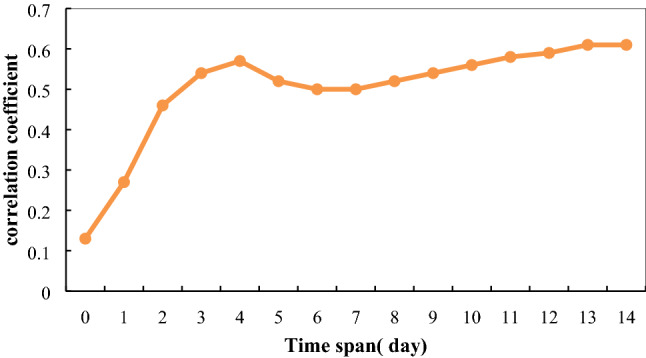
The correlation coefficients between Wuhan population flow and cumulative confirmed cases, excluding Hubei, considering different time intervals.

In China, COVID-19 was first reported from Wuhan, and most of the cases reported in other regions had connection to Wuhan (whether entered from Wuhan or had contact with Wuhan natives). The National Health Commission announced that the first confirmed case in Wuhan was on January 10, and Wuhan began to “close the city” on January 23^46^. Taking into account that the incubation period of the COVID-19 is around 0–14 days^1,21,47,48^, we calculated the correlation coefficients between the cumulative confirmed cases, excluding Hubei, and the population flow with the interval of 0–14 days from January 10 to February 6 (January 23 plus 14 days), with the interval of 0–14 days. As can be seen in Fig. 5, as the time span increased, higher correlation coefficients were observed and the correlation reached the first peak when we considered an interval of 4 days. From our results, it can be inferred that the effective time span for considering the influence of Wuhan Population flow on epidemic is 4 days. This is more detailed than those studies that have claimed the interval of 3–7 days for taking into account the influence of population flow^1,21,48^.

**Figure 6:**
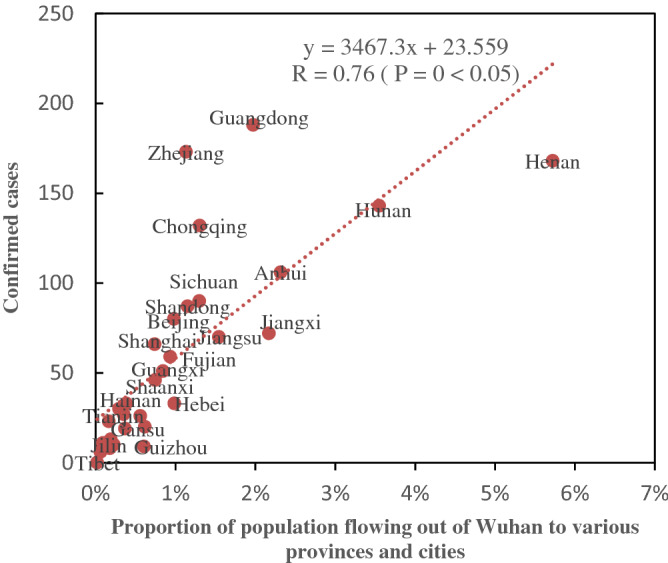
Correlation between the number of confirmed cases in different provinces and the population flow of Wuhan.

Figure 6 shows the linear relationship between the cumulative number of confirmed cases in each region and the population flow of Wuhan on January 27 (interval of 4 days). The results showed a high positive correlation (r = 0.76) between cumulative confirmed cases and population flow, indicating that the COVID-19-infected population entered from Wuhan had great influence on epidemic and spread of the virus in other parts of China.

On January 27, the top ten provinces based on the number of confirmed cases were Guangdong, Zhejiang, Henan, Hunan, Chongqing, Anhui, Sichuan, Shandong, Beijing and Jiangxi. The provinces with the highest population flow from Wuhan are Henan, Hunan, Anhui and Jiangxi, all of which are neighboring provinces in Hubei province. Consequently, it can be said that the closer they are to Hubei, the greater the population movement. This finding is consistent with the first law of geography that states similar things are more related than distant things. At the same time, Guangdong and Zhejiang, as two economically well-developed regions, had high number of confirmed cases. Although these two provinces are not neighbors of Wuhan, because of their strong economy, they have high population flow from Wuhan. Therefore, we can infer that those provinces with high number of confirmed cases had higher number of people who had enter from Wuhan. This finding suggests that beside geographical distance, the economic conditions also have influence on population flow and as result the distribution of the virus.

#### Analysis of the impact of prevention and control measures

**Figure 7:**
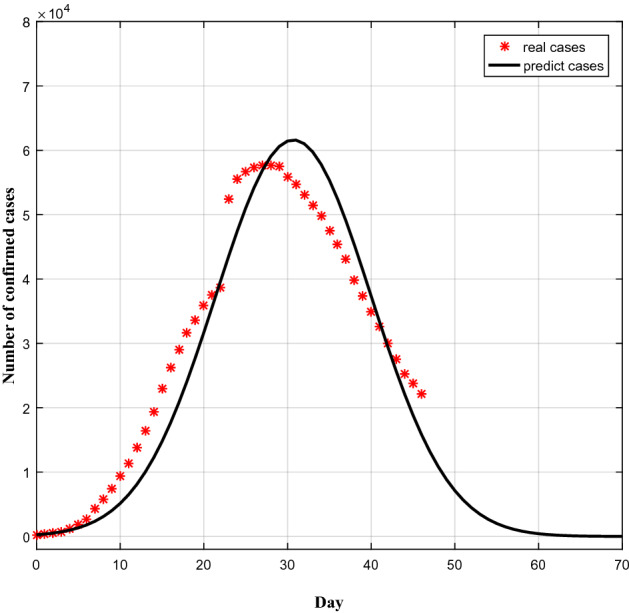
Comparison between observed and predicted prevalence curve of COVID-19.

Since the outbreak of COVID-19, in response to epidemic, the Chinese government has introduced a series of prevention and control measures such as restricting the activities and business that involve gathering people together, and many cities have been on lockdown (at different levels of severity). As it was discussed in "SIR model" section, based on regression analysis, the parameters of the time function were estimated as: a = − 0.0058 and b = 0.4276. Consequently, the infection factor in China declines by time, indicating that the prevention and control measures in China are efficient to control the epidemic. Based on available data and the estimated parameters *a* and *b*, we used an improved SIR model to predict the prevalence curve of COVID-19 in China (Fig. 7). As can be seen, the predicted number of patients is in line with the actual number of patients. Based on the predicted curve, the outbreak was predicted to reach a turning point on day 31, i.e. the outbreak reached an inflection point on February 20. Considering the actual inflection was observed on February 17, it can be inferred that our model is reliable.

**Figure 8:**
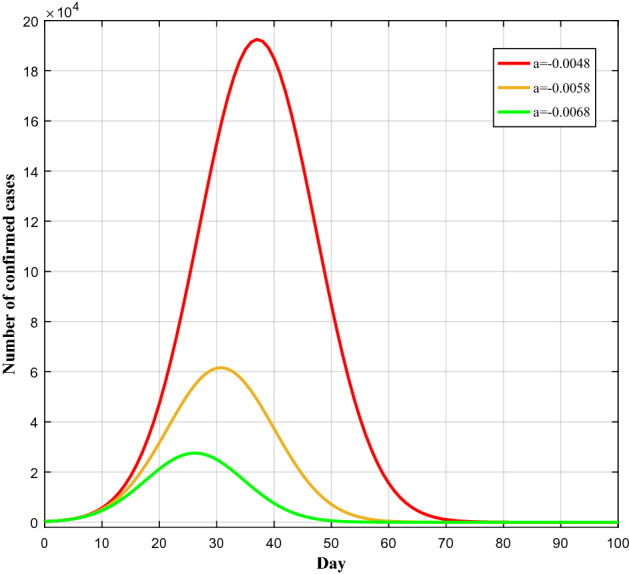
Prediction of the number of cases under different infection factors.

The intensity of prevention and control measures has direct effect on reduction of exposure to virus and the length of the epidemic period. To investigate the prevalence under different prevention and control measures, we simulated the COVID-19 prevalence curve considering different values of prevention intensity (*a*). As shown in Fig. 8, the trend of COVID-19 outbreak, predicted by our model, varies remarkably under different NPIs intensities. The more intensive controls lead to lower COVID-19 peak infection rate and decrease more the spread of outbreak. The simulated curve with a = − 0.0058 is similar to the observed prevalence curve of China, and shows a peak of 61,586 cases on day 31. With the less intensive interventions, a = − 0.0048, a peak of 192,495 cases can be observed on day 37 that is 2.1 times more than the cases with a = − 0.0058, and the epidemic lasts longer as well. This highlights the role of NPIs in control of the epidemic. With less intensive NPIs in China, the epidemic could last longer and more people get infected. Therefore, the control measures accomplished by China were effective. However, with more intensive prevention and control measures, a = − 0.0068, a peak of 27,566 cases is obtained on day 26 which is almost 55% less than the peak value in a = − 0.0058. This indicates that, if the NPIs in China started earlier or with more intensity, the number of infected cases would be decreased considerably. From our results, it can be inferred that larger absolute value of prevention intensity refers to the more intensive controls which lead to lower number of confirmed cases. Moreover, the duration of the epidemic has direct relation with prevention and controlling measures and selection of prevention intensity. China's successful interventions to contain the epidemic can be used as a reference for combating COVID-19 and even other respiratory-borne diseases around the world. In addition, to sustainably control the spread of virus, it is suggested that the governments decrease the intensity of the NPIs gradually.

## Conclusions

At the end 2019, COVID-19 was first reported in Wuhan. Thereafter, the number diagnosed cases increased rapidly, and the WHO announced COVID-19 as a global pandemic. In this study, we investigated the characteristics and main influencing factors of COVID-19. The main conclusions are as following:

1. The daily new confirmed cases of COVID-19 in China from 21 January 2020 to 12 March 2020 were consistent with the trend of daily new confirmed cases in Hubei Province. The daily new confirmed cases in non-Hubei Province, according to the spread of the epidemic, could be divided into five stages. In stage II the new peak of the epidemic was observed, and stage V led to level off.
2. Considering the spatial characteristics, the confirmed cases of COVID-19 were observed in all provinces, with wide ranges of distributions. There were differences in prevalence, recovered rates and mortality rates in all provinces. The cumulative confirmed cases in the provinces around Hubei Province or provinces with high population flow were higher than those in other provinces, especially those in the northwest.
3. Considering the local spatial autocorrelation, the cumulative confirmed cases in cities outside Hubei Province on March 12 have significant positive spatial autocorrelation, showing the spatial aggregation pattern, and the hot spots were concentrated in the cities of the southeastern provinces of Hubei Province.
4. Analysis of 31 cities showed that the cumulative confirmed cases of COVID-19 were significantly positively correlated with temperature (i.e., average daily temperature, daily maximum temperature and daily minimum temperature), relative humidity and precipitation during the study period.
5. The correlation coefficient between population flow of Wuhan and number of confirmed cases was high (r = 0.76). Moreover, a period of 4 days was observed between cumulative confirmed cases in other provinces and the population flow entered from Wuhan.
6. Based on the SIR model, our predicted prevalence curve of COVID-19 was compatible with observed curve of China. There is a large difference in the number of COVID-19 cases under different prevention and control measures. The simulated curve with a = − 0.0058 is similar to the real prevalence curve of China, and shows a peak of 61,586 cases on day 31.

Despite the important findings of this study, there are some limitations of the present study. First, in terms of data sources, although the China Health Commission makes daily announcements on COVID-19 data, the actual number of patients generally exceeds the number of registered patients. There are reports that the official early-announced data do not include asymptomatic cases, which will affect the accuracy of the analysis. Second, since the population data of each province in China during the epidemic is not available, the prevalence calculated using the data in the “China Statistical Yearbook 2019”. Third, limited access to more detailed data (infected cases, social, economic, and natural factors) that prevent us to have more comprehensive understanding about COVID-19 outbreak in China. Therefore, considering more datasets in the future studies, will enhance our understanding about COVID-19 epidemics and its influencing factors.

In summary, the spatial analysis of COVID-19 outbreak at national level showed positive spatial autocorrelation with hotspots in southeast of Hubei province, Taiwan and inner Mongolia. Moreover, temperature (including daily average temperature, daily maximum temperature, daily minimum temperature and diurnal temperature variation), precipitation and relative humidity had relationship with COVID-19 epidemics. Considering the social factor, there was a significant positive correlation between population outflow from Wuhan and the outbreak. The results of SIR model in this study showed compatibility with observed results that confirms the feasibility of this model in analyzing the prevention and control measures implemented by government. Moreover, our simulation results revealed the importance of intensive NPIs in controlling the epidemic. The findings and methods of this study could help for understanding different influencing factors of COVID-19 and aid governments and decision makers to simulate the results of different prevention and control measures.
